# Drastic improvement of Curie temperature by chemical pressure in *N*-type diluted magnetic semiconductor Ba(Zn,Co)$_{2}$As$_{2}$

**DOI:** 10.1038/s41598-021-86205-2

**Published:** 2021-04-07

**Authors:** Licheng Fu, Yilun Gu, Guoxiang Zhi, Haojie Zhang, Rufei Zhang, Jinou Dong, Xueqin Zhao, Lingfeng Xie, Fanlong Ning

**Affiliations:** 1 Zhejiang Province Key Laboratory of Quantum Technology and Device and Department of Physics, Zhejiang University, Hangzhou, 310027 China; 2 Collaborative Innovation Center of Advanced Microstructures, Nanjing University, Nanjing, 210093 China

**Keywords:** Magnetic properties and materials, Semiconductors, Spintronics

## Abstract

We report the effect of chemical pressure on the ferromagnetic ordering of the recently reported *n*-type diluted magnetic semiconductor Ba(Zn$_{1-x}$Co$_{x}$)$_{2}$As$_{2}$ which has a maximum $T_C$ $\sim$ 45 K. Doping Sb into As-site and Sr into Ba-site induces negative and positive chemical pressure, respectively. While conserving the tetragonal crystal structure and *n*-type carriers, the unit cell volume shrink by $\sim$ 0.3$\%$ with 15$\%$ Sr doping, but drastically increase the ferromagnetic transition temperature by 18$\%$ to 53 K. Our experiment unequivocally demonstrate that the parameters of Zn(Co)As$_{4}$ tetrahedra play a vital role in the formation of ferromagnetic ordering in the Ba(Zn,Co)$_{2}$As$_{2}$ DMS.

## Introduction

Diluted magnetic semiconductors (DMSs) that combine the properties of semiconductors and ferromagnets are promising materials for spintronic devices owing to the possible manipulation of both spin and charge degrees of freedom^1–4^. In 1990s, based on the non-equilibrium growth conditions of low temperature Molecular Beam Epitaxy (LT-MBE) method, some DMSs thin films, such as (Ga,Mn)As and (In,Mn)As, were successfully prepared and show hole-induced ferromagnetic ordering^5,6^. Among them, (Ga,Mn)As is one of many well investigated DMSs and it has been realized many spintronic functionalities in the last three decades^7–9^. In (Ga,Mn)As, spins and holes are introduced by Mn$^{2+}$/Ga$^{3+}$ substitutions simultaneously. The highest $T_C$ obtained in (Ga,Mn)As is $\sim$ 200 K when Mn$^{2+}$ doping level reaches $\sim$ 12$\%$^10^, which is still below room temperature that are required for practical applications. On the other hand, high temperature ferromagnetism with *n*-type carriers has been reported in Fe-doped (In,Fe)As and (In,Fe)Sb films, the Fe atoms play the role of local magnetic moments, while free carriers are induced separately by codoped nonmagnetic donors/acceptors or native defects^11–13^.

In recent years, some new series of bulk form DMSs, that are iso-structure to the iron-based superconductors were reported. Such as 111-type Li(Zn,Mn)As^14^, Li(Zn,Mn)P^15^, 1111-type (La,Ba)(Zn,Mn)AsO^16^ and 122-type (Ba,K)(Zn,Mn)$_{2}$As$_{2}$^17,18^, which are iso-structure to the iron-based superconductors LiFeAs^19^, LaFeAsO$_{1-\delta }$^20^ and (Ba,K)Fe$_{2}$As$_{2}$^21^, respectively. In these new materials, spins and carriers are introduced at different ionic sites, which makes it possible to manipulate them separately, and investigate their unique contributions to the ferromagnetic ordering. In addition, the bulk form samples enable the utilization of some experimental techniques such as neutron scattering, nuclear magnetic resonance (NMR) and muon spin relaxation ($\mu SR$) that are usually based on bulk form materials^14–17,22–24^.

Among these new bulk form DMSs, the Curie temperature of (Ba,K)(Zn,Mn)$_{2}$As$_{2}$ has reached as high as $\sim$ 230 K^18^. This temperature is still below room temperature requested for practical applications. For the purpose of improving $T_C$, both physical pressure and chemical pressure effects on (Ba,K)(Zn,Mn)$_{2}$As$_{2}$ have been studied^25–27^. Unexpectedly, the results show that the Curie temperature is suppressed by both external physical pressure and chemical pressure. Recently, our group reported the successful synthesis of a new *n*-type DMS Ba(Zn$_{1-x}$Co$_{x}$)$_{2}$As$_{2}$^28^ iso-structure to the *p*-type (Ba,K)(Zn,Mn)$_{2}$As$_{2}$. The $T_C$ reaches $\sim$ 45 K for *x* = 0.04. $\mu SR$ measurements have confirmed that the ferromagnetic ordering in Ba(Zn,Co)$_{2}$As$_{2}$ is homogeneous and intrinsic. We wonder how lattice expansion or reduction will affect the ferromagnetism in Ba(Zn,Co)$_{2}$As$_{2}$ and what parameters affect the ferromagnetic ordering the most.

In this paper, we report the chemical pressure effect of Sr substitution for Ba and Sb substitution for As on the *n*-type DMS Ba(Zn$_{0.96}$Co$_{0.04}$)$_{2}$As$_{2}$ with $T_C$ $\sim$ 45 K. We introduce positive and negative chemical pressure through Sr/Ba and Sb/As iso-valent substitution, respectively, to study the chemical pressure effect on the ferromagnetic ordering in Ba(Zn,Co)$_{2}$As$_{2}$. We find that 15$\%$ Sr substitution for Ba drastically improve $T_C$ by 18$\%$ to 53 K while conserving the tetragonal structure and *n*-type carriers. Our experiment unequivocally demonstrate that the parameters of Zn(Co)As$_{4}$ tetrahedra play a vital role in the formation of ferromagnetic ordering in the Ba(Zn,Co)$_{2}$As$_{2}$ DMS.

## Results and discussion

### X-ray diffraction

**Figure 1:**
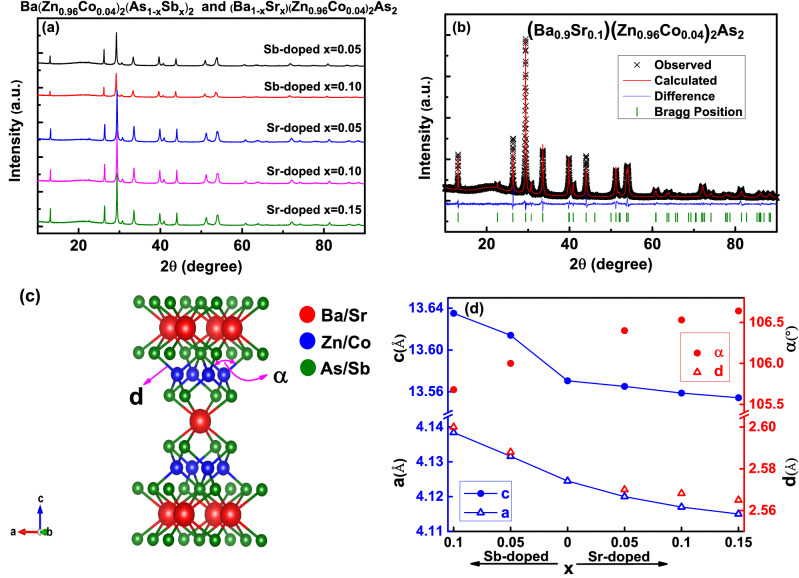
(**a**) The X-ray diffraction patterns for polycrystalline Sr-doped (Ba$_{1-x}$Sr$_{x}$)(Zn$_{0.96}$Co$_{0.04}$)$_{2}$As$_{2}$ (*x* = 0.05, 0.10 and 0.15) and Sb-doped Ba(Zn$_{0.96}$Co$_{0.04}$)$_{2}$(As$_{1-x}$Sb$_{x}$)$_{2}$ (*x* = 0.05 and 0.10). (**b**) The Rietveld refinement of (Ba$_{0.9}$Sr$_{0.1}$)(Zn$_{0.96}$Co$_{0.04}$)$_{2}$As$_{2}$. (**c**) The crystal structure of BaZn$_{2}$As$_{2}$ with tetragonal phase. The average As/Sb-Zn/Co band length is marked as *d* and the average As/Sb-Zn/Co-As/Sb bond angle is marked as $\alpha$ (bisected by *c* axis). (**d**) The lattice parameters, bond length *d* and bond angle $\alpha$ obtained from the Rietveld refinements for both Sb-doped and Sr-doped samples. The lattice parameters of Ba(Zn$_{0.96}$Co$_{0.04}$)$_{2}$As$_{2}$ are extracted from Fig. 1c of ref.^28^.

In Fig. 1a, we show polycrystalline X-ray diffraction patterns of Sr-doped (Ba$_{1-x}$Sr$_{x}$)(Zn$_{0.96}$Co$_{0.04}$)$_{2}$As$_{2}$ (*x* = 0.05, 0.10 and 0.15) and Sb-doped Ba(Zn$_{0.96}$Co$_{0.04}$)$_{2}$(As$_{1-x}$Sb$_{x}$)$_{2}$ (*x* = 0.05 and 0.10). We should mention that BaZn$_{2}$As$_{2}$ exhibit two different crystal structures, the low-temperature orthorhombic phase $\alpha$ -BaZn$_{2}$As$_{2}$ (space group Pnma)^29^ and the high-temperature tetragonal phase $\beta$-BaZn$_{2}$As$_{2}$ (space group I4/mmm) as a semiconductor with a bandgap $\sim$ 0.2 eV^30,31^. Both *p*-type DMS (Ba,K)(Zn,Mn)$_{2}$As$_{2}$ and *n*-type DMS Ba(Zn,Co)$_{2}$As$_{2}$ were achieved in the high temperature tetragonal phase^17,28^. The X-ray diffraction patterns for all samples can be well indexed by the tetragonal $\beta$-BaZn$_{2}$As$_{2}$ phase, and no orthorhombic $\alpha$-BaZn$_{2}$As$_{2}$ phase exist. That is, neither positive nor negative chemical pressure change the tetragonal crystal structure. In Fig. 1b, as an example, we show the Rietveld refinement of (Ba$_{0.9}$Sr$_{0.1}$)(Zn$_{0.96}$Co$_{0.04}$)$_{2}$As$_{2}$ with tetragonal $\beta$-BaZn$_{2}$As$_{2}$ phase using an open-source package GSAS-II^32^. The resultant weighted reliable factor R$_{wp}$ is $\sim$ 7.9 %. The crystal structure of the tetragonal phase is shown in Fig. 1c, which can be viewed as [ZnAs] layers(consist of ZnAs$_{4}$ tetrahedra) stacking alternately with Ba layers along the *c* axis. In Fig. 1d, we show the lattice parameters, the averaged (As$_{1-x}$Sb$_{x}$)-(Zn$_{0.96}$Co$_{0.04}$) band length *d* and the averaged (As$_{1-x}$Sb$_{x}$)-(Zn$_{0.96}$Co$_{0.04}$)-(As$_{1-x}$Sb$_{x}$) bond angle $\alpha$ obtained from the Rietveld refinements for different doping levels. We can see that for Sb-doped samples, lattice parameters *a*, *c* and bond length *d* increase monotonically with the increasing of doping levels; This is because the atomic radius of As and Sb are $1.25\,\AA$ and $1.33\,\AA$, respectively. Substitution of Sb for As therefore produce a negative chemical pressure. On the other hand, the ionic radius of Ba$^{2+}$ and Sr$^{2+}$ are $1.35\,\AA$ and $1.13\,\AA$, respectively. Therefore, substitution of Sr for Ba decrease *a*, *c* and the bond length *d*, which produce positive chemical pressure. For a non-distorted ideal tetrahedron, bond angle should be 109.47$^{\circ }$. In our samples, with more Sb doping, $\alpha$ tends to deviate from the ideal value; while with more Sr doping, the $\alpha$ tends to be more close to the value of 109.47$^{\circ }$.

### Magnetic properties

**Figure 2:**
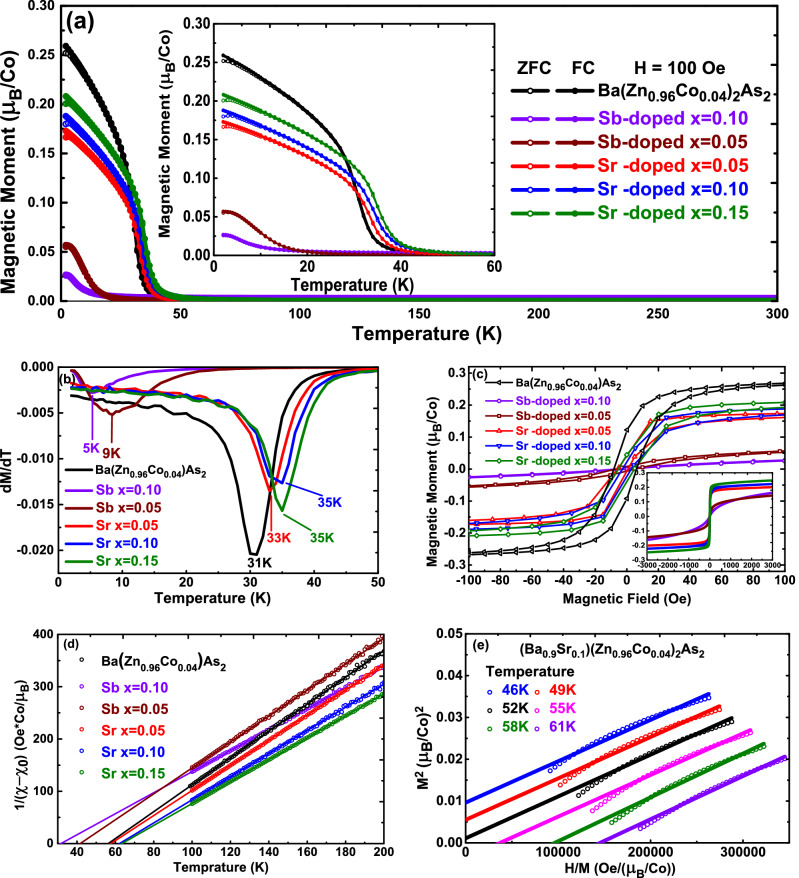
(**a**) Temperature dependent magnetization for Sb-doped and Sr-doped samples in zero-field cooling (ZFC) and field cooling (FC) conditions with an applied external field of 100 Oe. Inset shows the partial enlarged curves with temperature from 2 to 60 K. The M-T data of Ba(Zn$_{0.96}$Co$_{0.04}$)$_{2}$As$_{2}$ extracted from Fig. 3a of ref.^28^; (**b**) The *dM*(*T*)/*dT* versus *T* curves. (**c**) The isothermal magnetization curves for both Sb-doped and Sr-doped samples at 2 K. Inset shows the full range from − 3000 to 3000 Oe; (**d**) $(\chi -\chi _{0})^{-1}$ versus temperature plots with temperature range from 100 to 200 K, straight lines show the Curie-Weiss fit. (**e**) The Arrot-Noakes plots measured with magnetic field H from 1 to 5 T and the linear-fitting in high field region for (Ba$_{0.9}$Sr$_{0.1}$)(Zn$_{0.96}$Co$_{0.04}$)$_{2}$As$_{2}$.

In Fig. 2a, we show the temperature dependent magnetization for both Sb-doped and Sr-doped samples in zero-field cooling (ZFC) and field cooling (FC) conditions with an applied external field of 100 Oe. No significant magnetic transition are observed above 60 K. Abrupt increase of magnetization takes place at lower temperature, indicating the ferromagnetic transition. Compared to the Ba(Zn$_{0.96}$Co$_{0.04}$)$_{2}$As$_{2}$ whose $T_C$ is 45 K, we can see that the transition moves to lower temperature region with Sb doping while moves to higher temperature region with Sr doping. This trend can also be seen in the plot of *dM*(*T*)/*dT* versus *T*, as shown in Fig. 2b. The minimum value of *dM*/*dT* decreases with Sb doping, and increases with Sr doping. In Fig. 2c, we show the isothermal magnetization curves for both Sb-doped and Sr-doped samples at 2 K. Clear hysteresis loops demonstrate the ferromagnetic ordering state with the coercive field $\sim$ 10 Oe, which is similar to the Ba(Zn$_{0.96}$Co$_{0.04}$)$_{2}$As$_{2}$^28^.

We fit the paramagnetic part of the temperature dependent magnetization curves with a modified Curie-Weiss formula, $(\chi -\chi _{0})^{-1}=(T-\theta )/c$, $\chi _{0}$ is a temperature-independent component, *C* is the Curie constant and $\theta$ is the Weiss temperature. In Fig. 2d, we show the $(\chi -\chi _{0})^{-1}$ versus temperature plots with temperature range from 100 to 200 K, much higher than the ferromagnetic transition temperature. Through linear-fitting, we can get Weiss temperature $\theta$ from the intersection of the linear-fit lines and *x* axis. The effective moment $\mu _{eff}$ can also be obtained by using formula $C=N\mu _{0}\mu _{eff}^{2}/3K_{B}$. Furthermore, to determine the Curie temperature ($T_C$) accurately, Arrot-Noakes plots^33,34^ (through redraw the iso-thermal magnetization curves as $M^{2}$ versus *H*/*M*) were applied to the Sr-doped samples. Around $T_C$, the data points should form a series of parallel lines in the high field region; while at $T_C$, the parallel line should pass through the origin. In Fig. 2e, we show the Arrot-Noakes plots and the linear-fitting in high field region for (Ba$_{0.9}$Sr$_{0.1}$)(Zn$_{0.96}$Co$_{0.04}$)$_{2}$As$_{2}$, the $T_C$ is identified as 52 K.

In Table 1, we list all these parameters obtained above. Comparing with those of Ba(Zn$_{0.96}$Co$_{0.04}$)$_{2}$As$_{2}$^28^, we can see that the $T_C$ decreases with negative chemical pressure induced by Sb-doping, similar behavior has also been observed in Sb-doped *p*-type (Ba,K)(Zn,Mn)$_{2}$As$_{2}$^25^. While for Sr-doped samples, which provide positive chemical pressure, $T_C$ moves to the high temperature region. This is opposite to the case observed in (Ba,K)(Zn,Mn)$_{2}$As$_{2}$, where positive chemical pressure induced by P/As substitution suppress the $T_C$^25^. Previous results have shown that long-range ferromagnetic interaction in many *p*-type DMSs is predominantly mediated by the itinerant carries^26,27,35–39^. In *p*-type (Ba,K)(Zn,Mn)$_{2}$As$_{2}$, the long-range magnetic ordering is mediated by the *p* states of As through As 4*p*-Mn 3*d* hybridization. Shortened Zn/Mn-As bond length and optimized As-Zn/Mn-As bond angle (109.47$^{\circ }$ for an ideal tetrahedron) will enhance this *p*-*d* hybridization and the indirect exchange interaction between Mn dopants. Applying physical pressure to (Ba,K)(Zn,Mn)$_{2}$As$_{2}$ will decrease Zn/Mn-As bond length and drive the As-Zn/Mn-As bond angle away from 109.47$^{\circ }$. Therefore, $T_C$ is suppressed^27^. In *n*-type Ba(Zn,Co)$_{2}$As$_{2}$, with the same crystal structure, as shown in Fig. 1d, the bond length *d* increases and the bond angle $\alpha$ deviates from the ideal value with Sb-doping. While for Sr-doped samples, instead of the direct influence on the Zn(Co)As$_{4}$ tetrahedra with substitution on As sites, the Sr/Ba substitution manipulates the lattice parameters and then moderately shortens the bond length *d* and drive the bond angle $\alpha$ close to the ideal value of 109.47$^{\circ }$. We conclude that the parameters of Zn(Co)As$_{4}$ tetrahedra plays an important role in the formation of ferromagnetic ordering in *n*-type Ba(Zn,Co)$_{2}$As$_{2}$ DMS, the more (As$_{1-x}$Sb$_{x}$)-(Zn$_{0.96}$Co$_{0.04}$)-(As$_{1-x}$Sb$_{x}$) bond angle $\alpha$ closer to 109.47$^{\circ }$, the higher Curie temperature can be achieved.

**Table 1:** $T_{dif}$ from the minimum value of dM/dT, Weiss temperature $\theta$ from Curie-Weiss fit, Curie temperature $T_C$ from Arrot-Noakes plots and the effective moment $\mu _{eff}$ for different doping levels *x*.

Doping level *x*	$T_{dif}$ (K)	$\theta$ (K)	$T_C$ (K)	$\mu _{eff}$ ($\mu _{B}$/Mn)
Sb-doped 0.10	5	32	–	1.3
Sb-doped 0.05	9	42	–	1.4
Ba(Zn$_{0.96}$Co$_{0.04}$)$_{2}$As$_{2}$^28^	31	57	45	1.4
Sr-doped 0.05	33	59	49	1.4
Sr-doped 0.10	35	62	52	1.4
Sr-doped 0.15	35	63	53	1.4

### Transport and Hall effect

**Figure 3:**
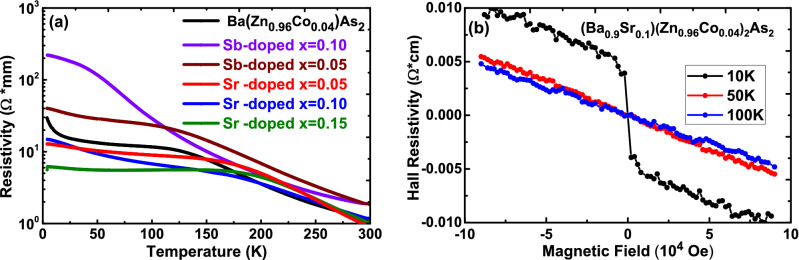
(**a**) Temperature-dependent resistivity of Ba(Zn$_{0.96}$Co$_{0.04}$)$_{2}$As$_{2}$ (extracted from Fig. 5 of ref^28^), Sr-doped and Sb-doped samples. (**b**) Hall resistivity of (Ba$_{0.9}$Sr$_{0.1}$)(Zn$_{0.96}$Co$_{0.04}$)$_{2}$As$_{2}$ at different temperatures, demonstrating *n*-type carriers.

In Fig. 3a, we show the temperature-dependent resistivity of Sr-doped and Sb-doped Ba(Zn$_{0.96}$Co$_{0.04}$)$_{2}$As$_{2}$ samples, respectively. For all studied samples, the resistivity increases with decreasing temperature, indicating that our samples retain semiconducting behavior under either positive or negative chemical pressure. For Sb-doped sample, the resistivity quickly increases with decreasing temperature and is much higher than that of Ba(Zn$_{0.96}$Co$_{0.04}$)$_{2}$As$_{2}$ in low temperature region. While in contrast, the resistivity of Sr-doped sample is lower than that of Ba(Zn$_{0.96}$Co$_{0.04}$)$_{2}$As$_{2}$ in most temperature range. This can be attribute to the broadened electronic bandwidth and increased carrier mobility with compression of the lattice^26,35^. To examine whether the type of carriers for Sr-doped samples with iso-valent doping has been changed, we measured Hall effect. We show (Ba$_{0.9}$Sr$_{0.1}$)(Zn$_{0.96}$Co$_{0.04}$)$_{2}$As$_{2}$ as an example in Fig. 3b. The negative slope of the Hall resistivity at different temperatures demonstrates that the dominant carriers are electrons and the carrier concentration is roughly estimated to be $\sim$ 10$^{18}$/cm$^{-3}$, comparable to Ba(Zn$_{0.96}$Co$_{0.04}$)$_{2}$As$_{2}$^28^. These results demonstrate that positive chemical pressure induced by Sr/Ba substitution increases the ferromagnetic transition temperature $T_C$ and electrons are still dominant carriers.

## Conclusion

To conclude, we have successfully synthesized both Sr-doped (Ba$_{1-x}$Sr$_{x}$)(Zn$_{0.96}$Co$_{0.04}$)$_{2}$As$_{2}$ and Sb-doped Ba(Zn$_{0.96}$Co$_{0.04}$)$_{2}$(As$_{1-x}$Sb$_{x}$)$_{2}$ DMSs via solid-state reaction method. The X-ray diffraction measurements confirm that both Sr-doped and Sb-doped samples retain the tetragonal crystal structure. Hall effect measurements show that the dominant carriers are still electrons for Sr-doped sample. Magnetization measurements reveal that the ferromagnetic transition temperature $T_C$ decreases with Sb-doping while increases by 18$\%$ to 53 K with 15$\%$ Sr-doping. Comparing with the results of *p*-type (Ba,K)(Zn,Mn)$_{2}$As$_{2}$^25,27^, we find that the parameters of Zn(Co)As$_{4}$ tetrahedra have great influence on the formation of ferromagnetic ordering. Our work shows an effective method to modify the magnetic properties of *n*-type DMS Ba(Zn,Co)$_{2}$As$_{2}$ via proper chemical pressure and offers a good example for further experimental,computational and theoretic investigations about the mechanism of the ferromagnetic ordering in bulk form diluted magnetic semiconductors.

## Methods

### Material synthesis

Polycrystalline samples Sr-doped (Ba$_{1-x}$Sr$_{x}$)(Zn$_{0.96}$Co$_{0.04}$)$_{2}$As$_{2}$ (*x* = 0.05, 0.10 and 0.15) and Sb-doped Ba(Zn$_{0.96}$Co$_{0.04}$)$_{2}$(As$_{1-x}$Sb$_{x}$)$_{2}$ (*x* = 0.05 and 0.10) were prepared by conventional solid-state reaction method, similarly to that of Ba(Zn,Co)$_{2}$As$_{2}$^28^. High purity Ba, Sr, Zn, Co, As, Sb elements were mixed and placed in alumina crucibles and sealed in evacuated silica tubes. The mixture was heated at 1150 $^{\circ }$C for 25 h before cooling to room temperature. The products were then grounded, pressed into pellets, sealed in evacuated silica tubes again and reheated at 1150 $^{\circ }$C for another 25 h for further reaction. Then they were quickly cooled to room temperature, the high-temperature phase, tetragonal $\beta$-BaZn$_{2}$As$_{2}$^40^ can be obtained.

### Experimental characterization

The crystal structure of the polycrystalline samples were measured at room temperature using a PANalytical powder X-ray diffractometer with monochromatic Cu-K$_{\alpha 1}$ radiation. The DC magnetization measurements were conducted on a Quantum Design Magnetic Property Measurement System (MPMS). The Hall effect were measured using a Quantum Design Physical Property Measurement System(PPMS). The electrical resistivity was measured on sintered pellets using the typical four-probe method.

## Data Availability

All data generated or analysed during this study are included in this published article or available from the corresponding author on reasonable request.
